# Femtosecond Laser Processing Assisted SiC High-Temperature Pressure Sensor Fabrication and Performance Test

**DOI:** 10.3390/mi14030587

**Published:** 2023-02-28

**Authors:** You Zhao, Yulong Zhao, Lukang Wang, Yu Yang, Yabing Wang

**Affiliations:** State Key Laboratory for Manufacturing Systems Engineering, Xi’an Jiaotong University, Xi’an 710049, China

**Keywords:** SiC, high temperature, pressure sensor, fabrication method, feasibility

## Abstract

Due to material plastic deformation and current leakage at high temperatures, SOI (silicon-on-insulator) and SOS (silicon-on-sapphire) pressure sensors have difficulty working over 500 °C. Silicon carbide (SiC) is a promising sensor material to solve this problem because of its stable mechanical and electrical properties at high temperatures. However, SiC is difficult to process which hinders its application as a high-temperature pressure sensor. This study proposes a piezoresistive SiC pressure sensor fabrication method to overcome the difficulties in SiC processing, especially deep etching. The sensor was processed by a combination of ICP (inductive coupled plasma) dry etching, high-temperature rapid annealing and femtosecond laser deep etching. Static and dynamic calibration tests show that the accuracy error of the fabricated sensor can reach 0.33%FS, and the dynamic signal response time is 1.2 μs. High and low temperature test results show that the developed sensor is able to work at temperatures from −50 °C to 600 °C, which demonstrates the feasibility of the proposed sensor fabrication method.

## 1. Introduction

High-temperature pressure detection is widely needed in aerospace, nuclear power and the automobile industry [1]. For example, in aero-engine operation state evaluation, a pressure sensor is necessary for internal flow field motion monitoring [2,3], and the internal temperature of an aero-engine often exceeds 500 °C [4]; in the high-temperature reactor in nuclear power plants, the pressure change in high-temperature fluids (≥350 °C) in the pipeline needs real-time monitoring; in the combustion condition monitoring of an automobile engine, a high-temperature pressure sensor is also required (≥300 °C). Therefore, high-temperature pressure sensors are important and necessary for equipment performance tests and operational health monitoring [5,6]. However, current pressure sensors face some problems at high temperatures and in harsh environment applications. For example, piezoelectric pressure sensors have high sensitivity and good frequency response, which are suitable for dynamic pressure signal measurement [7,8,9]. The 176A03 piezoelectric pressure sensor’s (developed by the American PCB company) working temperature range is −70–650 °C, and its frequency response is 10 kHz [10], which is suitable for internal engine pressure detection. However, piezoelectric sensors lack enough advantages in miniaturization and cost (because extra precise charge amplification is needed) for scale application. Capacitive pressure sensors have high resolution and low thermal sensitivity drift [11,12] and can also be miniaturized at low cost. For example, Chen Li reported a split-type pressure sensor based on differential capability with a working temperature range of 25–400 °C and a sensitivity of 9.27 mV/kPa [13,14]. However, capacitive pressure sensors can be affected by parasitic capacitance creating difficulties ensuring measurement accuracy in complex environments. The optical fiber pressure sensor is small in size, has anti-electromagnetic interference and high-temperature resistance [15,16] to overcome the above problems. For example, Fei Feng reported an optical fiber Fabry–Perot pressure sensor [17] as having a maximum linearity error of 1% in the temperature range of 20–400 °C; Mohammad Istiaque Reja developed a pure silica micro-structured optical fiber pressure sensor which can tolerate temperatures up to 800 °C [18]. However, the fiber Fabry–Perot cavity used for pressure sensing is sensitive to temperature changes, which causes unwanted output besides pressure change. In addition, a special light source, optical modulation and detection equipment are required for optical fiber pressure sensors, which are not convenient for use. Piezoresistive sensors are produced by micro and nano manufacturing technology, which is circumscribed as small size, high precision, low power consumption, simple measurement circuit and mass production [19,20], which is very suitable for integrated applications.

However, current piezoresistive pressure sensors are mainly based on silicon materials. Due to current leakage over 120 °C, the working temperature of silicon pressure sensors is limited. For example, the upper operating temperature limit of the 8515C piezoresistive pressure sensor developed by ENDEVCO Corporation is 125 °C [21]. Nonetheless, silicon-on-insulator (SOI) and silicon-on-sapphire (SOS) methods can alleviate the defects of high-temperature current leakage [22]. However, due to the plastic deformation of silicon at high temperatures (≥500 °C) and the thermal expansion mismatch of different materials, SOI and SOS pressure sensors usually fail when the temperature exceeds 500 °C.

Silicon carbide (SiC) is a good high-temperature resistant material, which keeps stable mechanical and electrical properties over 500 °C. It is a favorite choice to replace silicon for fabricating high-temperature pressure sensors, and researchers have reported many studies on SiC based high-temperature pressure sensors. For example, the NASA Glenn Research Center has successively reported sensors that can withstand 600 °C and 750 °C [23,24]; the optimal linearity error of the sensor at 600 °C is −0.14%. The Institute of Microelectronics of the Chinese Academy of Sciences has developed an all-SiC absolute pressure sensor through a grinding, chemical and mechanical polishing, inductively coupled plasma (ICP) etching and direct bonding method; research reveals that the sensor’s hysteresis, repeatability and nonlinearity are 0.171% FS, 0.232% FS and 0.034% FS, respectively, which is truly high precision [25]. In addition, they also studied the sensor’s thermal zero offset drift and the piezoresistive effect of SiC [26,27]. North University of China has also long worked on SiC high-temperature pressure sensors using reactive ion etching and inductively coupled plasma etching as the main processing methods [28]. The above studies have effectively proved the feasibility of SiC being used for high-temperature pressure sensors. However, due to high hardness, brittleness and corrosion resistance, SiC pressure sensor fabrication is very difficult, especially deep etching of the cavity structure. To obtain large etching depth and excellent processing quality, it often takes a lot of time and experiences many failures.

Therefore, finding a fast and effective processing method for SiC pressure sensors (especially fast etching with deep cavity structure) is of great significance. This study proposes a fabrication method for SiC high-temperature pressure sensors, including sensor design, fabrication and performance tests. The experiment result has demonstrated this fabrication method’s feasibility.

## 2. Sensor Design

**Figure 1:**
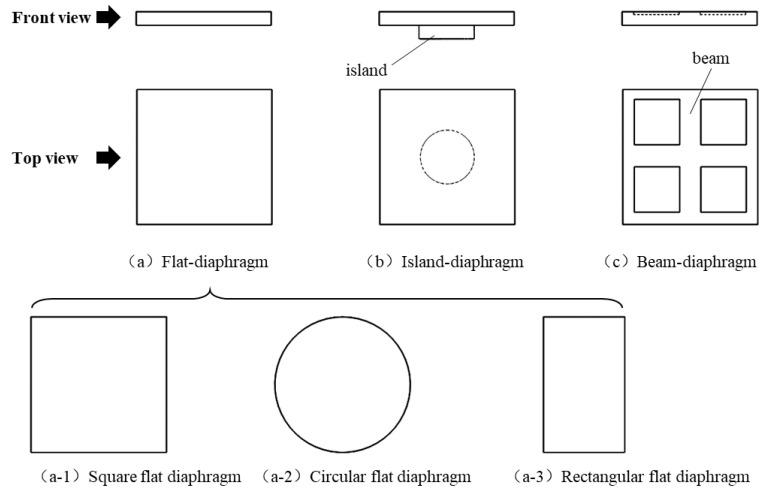
Common structures of pressure sensor’s sensitive diaphragm.

Piezoresistive pressure sensors often use a thin diaphragm as the pressure sensitive element. The sensitive diaphragm can be a flat diaphragm, island diaphragm or beam diaphragm, and the shape of the diaphragm can be circular, square or rectangular, as shown in Figure 1. For the island diaphragm and beam diaphragm, stress concentration occurs at the edge of the island or the beam, and it is conducive to sensitivity improvement and suitable for small range pressure measurement. For large range pressure measurement, the flat diaphragm is often used because stress distribution on the flat diaphragm is more uniform and lower than the island diaphragm and beam diaphragm.

In this study, a circular, flat diaphragm is chosen as the sensor’s sensitive diaphragm because (1) SiC is difficult to process due to its high hardness, high wear resistance and strong chemical inertia, and a simple structure is helpful in reducing the processing difficulty; (2) when the pressure measurement range is 0–5 Mpa, a circular, flat diaphragm is beneficial in reducing the stress generated on the diaphragm and improving safety.

**Figure 2:**
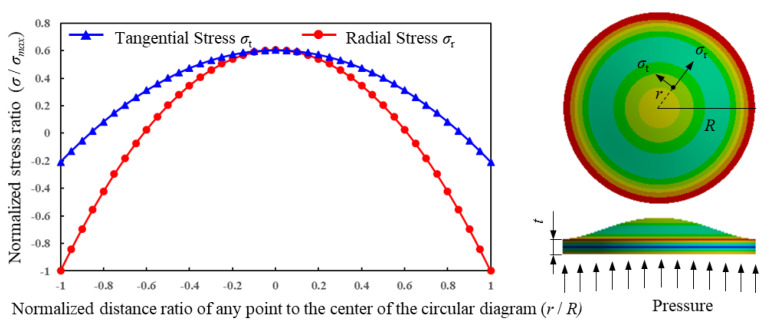
Stress variation curve and equivalent stress distribution on the circular, flat diaphragm.

According to the classical flat diaphragm theory, the surface stress calculation formulas of the circular, flat diaphragm with thickness *T* and radius *r* under the pressure *P* are

(1) $$\sigma_{r}=\frac{3P}{8t^{2}}\left[R^{2}\left(1+\mu \right)-r^{2}\left(3+\mu \right)\right]$$

(2) $$\sigma_{t}=\frac{3P}{8t^{2}}\left[R^{2}\left(1+\mu \right)-r^{2}\left(1+3\mu \right)\right]$$

where *σ_r_* and *σ_t_* represent the radial and tangential stress on the circular, flat diaphragm, *r* represents the distance from any position to the center of the diaphragm, and *μ* is Poisson’s ratio. Figure 2 illustrates the stress variation curve and equivalent stress distribution on the diaphragm.

**Figure 3:**
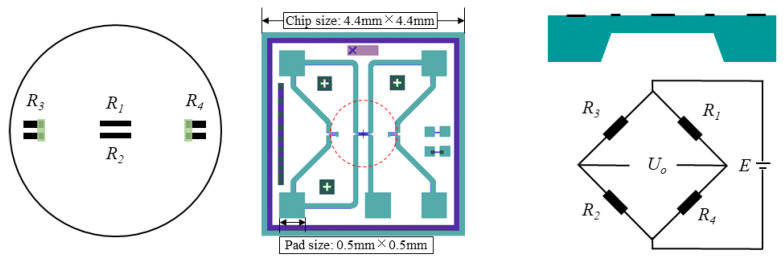
Structure of SiC pressure sensor and piezoresistor arrangement.

According to the literature [29], the longitudinal strain factor (*GF_l_*) of single crystal SiC on the (0001) crystal plane keeps the same in different crystal directions, and so does its transverse strain factor (*GF_t_*). For n-type 4H-SiC with a doping concentration of 1 × 10^19^ cm^−3^, its *GF_l_* and *GF_t_* is about −10 and 20.8, respectively. According to the stress distribution law for the circular, flat diaphragm in Figure 2, the structure of the SiC pressure sensor and piezoresistor arrangement on the circular, flat diaphragm was designed as shown in Figure 3. Specifically, the diameter and thickness of the circular, flat diaphragm is 1200 μm and 75 μm, respectively. Two single fold piezoresistors are arranged along the radial direction at the center of the circular diaphragm (size: 200 μm × 20 μm × 2 μm), and two double fold piezoresistors are arranged at both left and right edge of the diaphragm (size: 100 μm × 20 μm × 2 μm). For this kind of resistor arrangement, the absolute stress difference between the resistors is large, which is conducive to the differential output of the Wheatstone bridge. In addition, the piezoresistors’ layout scheme is in a radial direction which makes them deform mainly along their length, so the consistency of resistance change is better.

## 3. Sensor Fabrication

**Figure 4:**
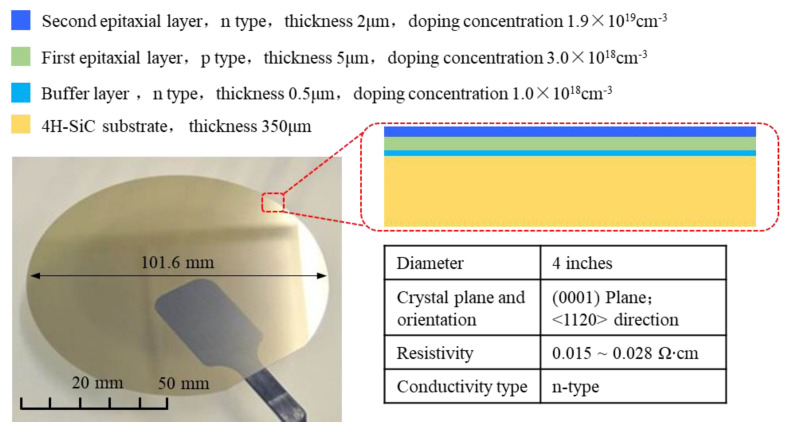
Structure and physical parameters of the 4H-SiC epitaxial wafer.

The sensor was fabricated using a 4-inch and n-type 4H-SiC epitaxial wafer. The structure and physical parameters of the wafer are shown in Figure 4.

**Figure 5:**
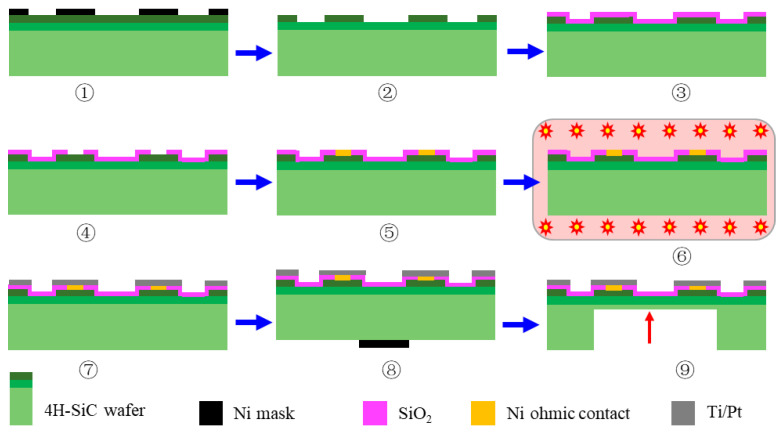
Schematic diagram of the SiC pressure sensor manufacturing process.

The SiC pressure sensor was fabricated mainly using MEMS (micro electromechanical system) technology and femtosecond laser deep etching technology. The main processing steps are shown in Figure 5: ① preparation of the ICP etching mask by metal sputtering, and a layer of 150 nanometer-thick nickel is sputtered on the wafer by an EXPLORE 14 magnetron sputtering system; ② fabrication of piezoresistors by ICP etching carried out on an Oxford Instruments plasma system100, and the etching depth should be higher than 2 μm; ③ depositing silica isolation layer (300 nm) on the wafer by the EXPLORE 14 magnetron sputtering system, which is used to protect the sensor from oxidation and acts as the ohmic contact window in the next step; ④ preparation of the ohmic contact window by lithography and wet etching, and silica in specific areas is corroded by hydrofluoric acid; ⑤ nickel is sputtered in the ohmic contact window for ohmic contact preparation in the next step; ⑥ ohmic contacts are formed by high-temperature rapid annealing around 950~1050 °C using an UNITEMP RTP-100 high-temperature rapid annealing furnace; ⑦ deposition of the Ti/Pt layer using the EXPLORE 14 magnetron sputtering system with a thickness of 500 nm; ⑧ fabrication of the back cavity position mark using lithography on an ABM/6 double-sided alignment exposure machine and metal sputtering on the EXPLORE 14 magnetron sputtering system; ⑨ back cavity deep etching using a femtosecond laser on a GCC-A4060 three-axis femtosecond laser micromachining system for about 250 μm, and then further etched by about 20 μm using ICP dry etching to smooth the cavity’s surface.

**Figure 6:**
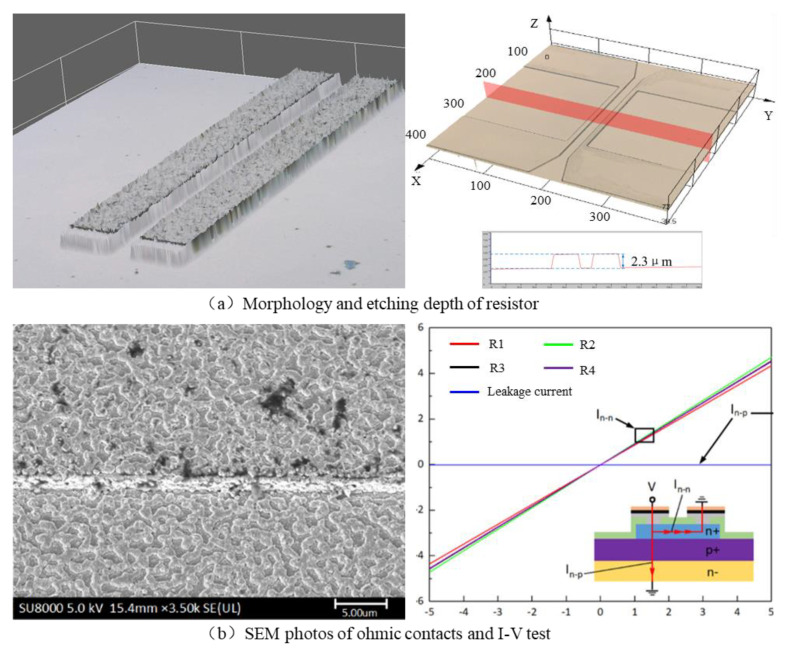
Key manufacturing process steps and test results of the SiC pressure sensor.

An Oxford Instruments plasma system100 was used for the ICP dry etching of the piezoresistors, and the average etching rate was about 200 nm/min. To ensure the insulation between the piezoresistors and peripheral SiC structure, the top n-type SiC epitaxial layer needs to be completely etched, and the step height of the ICP dry etching is 2.3 μm, as shown in Figure 6a.

SiC ohmic contact was generated by metallization annealing between 950 °C to 1050 °C. Metal Ni was selected as the contact layer, metal Ti was selected as the adhesion layer and metal Pt acted as the electrical connection layer. An UNITEMP RTP-100 high temperature rapid annealing furnace was used to prepare the ohmic contact. Figure 6b depicts the surface morphology of the ohmic contact area as well as its I-V test curves, and the measured contact resistivity was 9.7 × 10^−5^ Ω·cm^2^.

Due to the high hardness, good wear resistance and strong chemical inertia, conventional machining methods such as dry etching, wet etching and mechanical machining (ultrasonic assisted) are difficult to achieve in SiC deep etching. A femtosecond laser is featured with narrow pulse width, high energy, high frequency and negligible thermal effect, which is a kind of non-contact processing. It has the advantages of high flexibility (3 degrees of freedom), high speed and no material selectivity, which is very promising for the deep etching of SiC.

**Figure 7:**
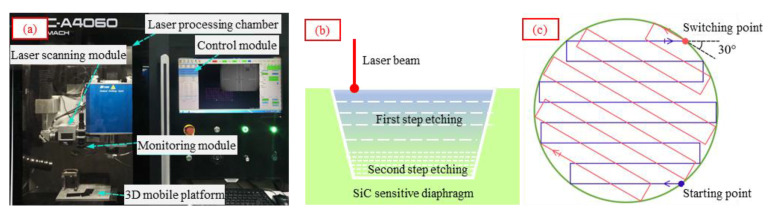
(**a**) A three-axis femtosecond laser micromachining system, (**b**) schematic diagram of the two-step laser etching of the SiC (**c**) and the schematic diagram of the laser scanning path.

In this study, a strategy of ICP dry etching assisted femtosecond laser deep etching was adopted. Firstly, the deep etching of the SiC cavity was completed using a GCC-A4060 three-axis femtosecond laser micromachining system, as shown in Figure 7a. A two-step laser etching method was used for the SiC deep cavity etching, as shown in Figure 7b,c, that is, the coarse etching of the SiC cavity using higher laser power, more scanning times and a larger scanning linewidth at first; a fine etching of the SiC cavity using a lower laser power, fewer scanning times and smaller scanning linewidth secondly. The average etching rate was 20 μm/min. The femtosecond laser etching parameters are shown in Table 1.

**Figure 8:**
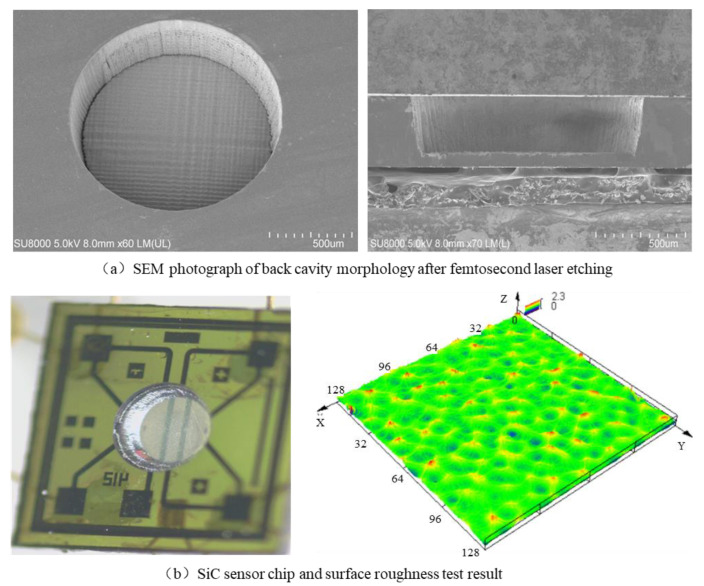
(**a**) SEM photograph of back cavity morphology after femtosecond laser etching, (**b**) SiC sensor chip and surface roughness test result.

Finally, the laser etched surface was further etched using ICP etching to reduce the roughness; the etching depth was 20–30 μm and the average etching rate was 0.8 μm/min. The total etching depth of the SiC cavity was 270–300 μm, and a circular, flat diaphragm with a thickness of 50–80 μm was obtained as shown in Figure 8, and the surface roughness of the diaphragm is 141 nm.

## 4. Results and Discussion

### 4.1. Static Calibration

**Figure 9:**
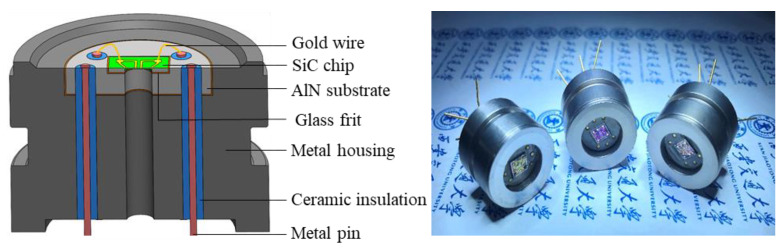
Gold wire bonding package of the SiC pressure sensor.

**Figure 10:**
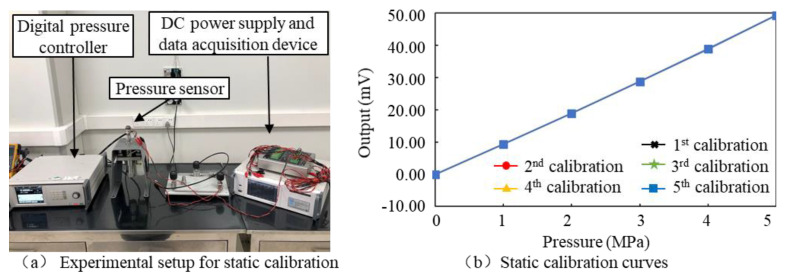
Experimental setup (**a**) and output curves (**b**) of static calibration.

The SiC pressure sensor was packaged by the gold wire bonding method. The schematic diagram of the gold wire bonding package and the packaged sensors are shown in Figure 9. Then, static calibration was carried out as shown in Figure 10a.

During static calibration, the pressure sensor was connected to a digital pressure controller and powered by 5 V DC voltage, and its output was collected by a parallel multi-channel pressure sensor data acquisition device. The loading pressure was 0 MPa, 1 MPa, 2 MPa, 3 MPa, 4 MPa and 5 MPa. The sensor was loaded from 0 MPa to 5 MPa and then unloaded from 5 MPa to 0 MPa during each calibration cycle; 5 cycles of calibration were carried out. The calibration results show that the accuracy error of the three developed sensors is 0.33%FS, 1.02%FS and 3.44%FS, respectively. The difference is mainly related to the sensor’s packaging process (the consistency of manual packaging varies a lot). For the sake of fairness, the sensor with an accuracy error of 1.02%FS is being taken for analysis and discussion. The static calibration results and performance indicators of the sensor are shown in Figure 10b, Table 2 and Table 3.

The experiment result shows that the accuracy of the developed SiC pressure sensor is 1.02% FS, and the repeatability error, hysteresis error and linearity error are 0.10% FS, 0.11% FS and 1.01% FS, respectively. Compared to other SiC pressure sensors in Table 2, the linearity error of the developed sensor is a little bit higher; on the one hand, this is related to the manual packaging of the sensors in this study, and due to the difficulties of ensuring the consistency of packaging effects that affects the performance of sensors; on the other hand, it is related to the deep etching quality of the femtosecond laser in this study. Compared with the sensitive diaphragm prepared using only ICP etching in other literature, the differences of the SiC rapid deep etching method in this paper are as follows:

(1) The surface roughness obtained using the SiC rapid deep etching method in this paper is higher: 141 nm vs. just a few nanometers or tens of nanometers in other literature. This causes non-uniformity of the surface stress distribution on the sensitive diaphragm, resulting in the inconsistent change of the piezoresistors and affecting the output linearity of the sensor.

(2) Femtosecond laser etching forms a groove at the edge of the sensitive diaphragm, which makes the stress change of the piezoresistors at the edge of the diaphragm inconsistent and is not linear with the change of pressure, causing the nonlinear output.

Two methods will be taken for improvement:

(1) Improving the femtosecond laser rapid deep etching parameters, reducing the surface roughness of the sensitive diaphragm and inhibiting the formation of the edge groove on the sensitive diaphragm.

(2) On the premise of low repeatability error and hysteresis error, using algorithm compensation to improve the linearity.

### 4.2. Dynamic Calibration

**Figure 11:**
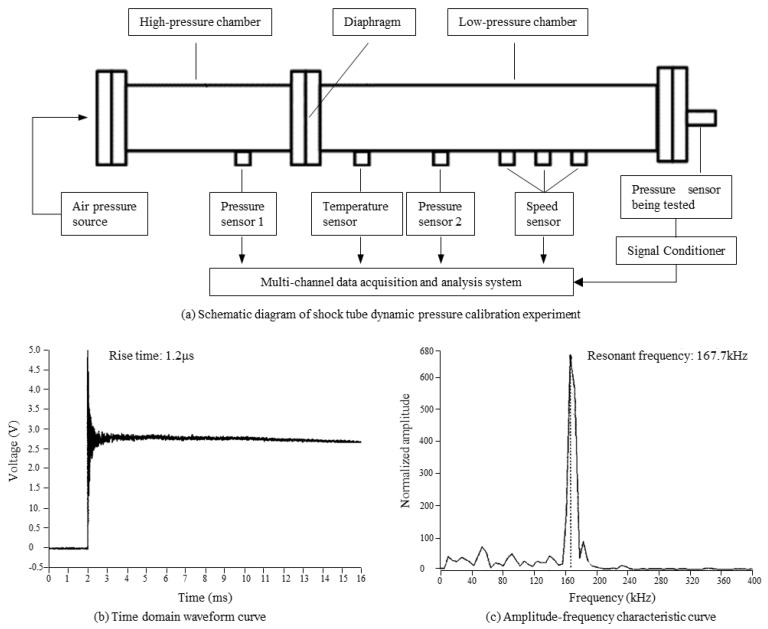
(**a**–**c**) Dynamic pressure calibration test and results.

A dynamic pressure calibration test was carried out to test the dynamic response ability of the sensor. A shock tube dynamic pressure standard device was used as the main experimental equipment. As shown in Figure 11a, the tested sensor was installed at the end of the shock tube, and the high-pressure chamber of the shock tube was pressurized using a high precision air pressure source. When the pressure difference between the high-pressure chamber and the low-pressure chamber reaches the pre-set value (for example, 3 MPa), the diaphragm between the high-pressure chamber and the low-pressure chamber breaks, then the gas in the high-pressure chamber rushes rapidly to the low-pressure chamber to form an incident shock wave forming a positive transient step pressure and acting on the tested sensor. The output of the tested sensor was collected using a multi-channel data acquisition and analysis system, and the frequency response of the tested sensor was obtained by the fast discrete Fourier transform.

The time domain waveform curve and amplitude frequency characteristic curve of the tested sensor were obtained through the dynamic calibration test, as shown in Figure 11b and Figure 10c, respectively. The sensor showed good response ability to the step signal with a rise time of 1.2 μs, and its resonant frequency was 167.7 kHz. Which means the sensor is able to measure high-frequency pressure signals in real-time.

### 4.3. High Temperature Performance Test

#### 4.3.1. Thermal Zero Offset Drift

**Figure 12:**
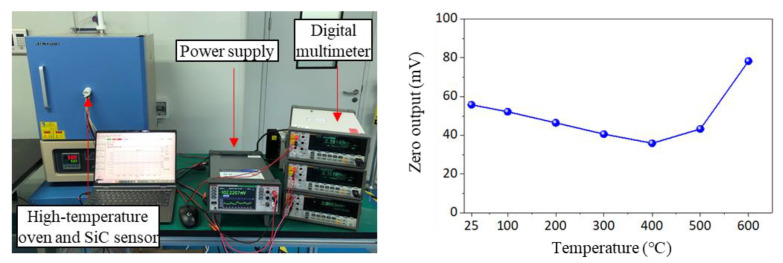
Experimental setup and zero offset drift curve.

The thermal zero offset drift test was taken to prove whether the sensor can survive high temperatures. The sensor was placed in a high-temperature oven, as shown in Figure 12. The testing temperature rose from 25 °C to 100 °C, 200 °C, 300 °C, 400 °C, 500 °C and 600 °C, with a rising rate of 1 °C/min, and each temperature was kept constant for 1.5 h. The zero offset of the sensor was recorded using a high-precision digital multimeter. Figure 11b depicts the zero offset drift curve of the sensor, and Table 4 illustrates the thermal zero offset drift coefficient (*TCO*) of each temperature according to Formula (3). In Formula (3), *Y*(*t*_1_) represents zero output of the sensor at 25 °C; *Y*(*t*_2_) represents zero output of the sensor at 100 °C, 200 °C, 300 °C, 400 °C, 500 °C and 600 °C; *Y_FS_* is full scale output of the sensor; *t*_1_ denotes a temperature of 25 °C; and *t*_2_ denotes temperatures of 100 °C, 200 °C, 300 °C, 400 °C, 500 °C and 600 °C.

(3) $$TCO=\frac{Y(t_{2})-Y(t_{1})}{Y_{FS}(t_{2}-t_{1})}\times 100\%$$

The *TCO* of the sensor was in the range of 0.01% to 0.06% from 25 °C to 600 °C, which was slightly optimized when compared to NASA (0.03~0.07%) [33]. In addition, it can be seen from Figure 11 that the zero output of the sensor kept decreasing from 25 °C to 400 °C, and then increasing when the temperature exceeded 400 °C.

This may relate to the change of residual stress inside the sensor because the SiC sensor was sintered with AlN substrate and metal housing through the glass frit, which experiences residual stress after cooling to room temperature. When the temperature rises from room temperature, the SiC chip and other materials tend to soften, and the residual stress on the sensor is partially released. Therefore, the sensor’s zero output also decreases. When the temperature continues to rise over 400 °C, the different thermal expansion between different materials becomes more and more obvious, resulting in increased stress on the sensor chip and increased zero output. This explanation is consistent with NASA’s research results [34].

#### 4.3.2. High and Low Temperature Calibration

**Figure 13:**
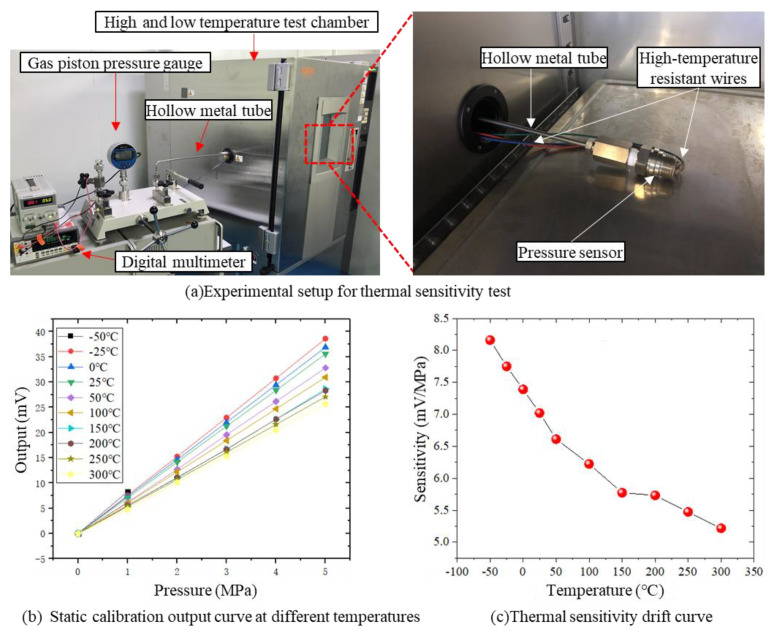
Experimental setup and results of thermal sensitivity drift test.

To further verify that the sensor can work at high and low temperatures, static calibration was carried out at different temperatures (−50 °C, −25 °C, 0 °C, 25 °C, 50 °C, 100 °C, 150 °C, 200 °C, 250 °C and 300 °C), as shown in Figure 13a. The sensor was placed in a high and low temperature test chamber, and high temperature-resistant wires were used to connect the sensor to both the power supply and a high-precision digital multimeter; a hollow metal tube was used to connect the sensor to a high-precision gas piston pressure gauge allowing the gas piston pressure gauge to apply standard pressure (0–5 MPa) to the sensor. Standard pressure (0 MPa, 1 MPa, 2 MPa, … 5 MPa) was applied to the sensor at different temperatures and the sensor’s output was recorded by the digital multimeter.

Figure 13b depicts the sensor’s output changing with the input pressure at different temperatures. The input pressure and output voltage of the sensor maintain a good linear relationship at different temperatures, indicating that the sensor can work normally in high and low temperatures. It can also be found that the sensitivity of the sensor decreases with the increase of temperature as shown in Figure 13c.

According to the pressure sensor’s sensitivity calculation Formula (4), its sensitivity is related to the piezoresistive coefficient of the SiC and the stress on the sensor chip. Where *Y_FS_* is full scale output of the sensor, *P* is the measuring range of the sensor, Δ*R* is the resistance change of the piezoresistor, *R*_0_ is the original resistance of the piezoresistor, *U*_0_ is the supply voltage, *π_l_* and *π_t_* present the longitudinal and transverse piezoresistive coefficients of SiC and *σ_l_* and *σ_t_* present the longitudinal and transverse stress on the piezoresistor.

(4) $$S=\frac{Y_{FS}}{P}=\frac{\frac{\Delta R}{R_{0}}U_{0}}{P}=\frac{\left(\pi_{l}\sigma_{l}+\pi_{t}\sigma_{t}\right)U_{0}}{P}$$

For piezoresistive sensors, the piezoresistive coefficient often decreases with the increase in temperature, resulting in the decrease of the sensitivity, and vice versa. This is consistent with the change of sensor sensitivity in this experiment. In addition, temperature changes will also cause changes in the size and Poisson’s ratio of sensor materials. On the one hand, the sensor chip is subject to thermal stress caused by material expansion, and on the other hand, it is subject to normal stress caused by pressure. The change of thermal stress and normal stress at different temperatures will also affect the sensitivity of the sensor, but the influence mechanism is complex which is beyond the scope of this article.

## 5. Conclusions

In order to break through the operating temperature upper limit of current silicon-based high-temperature pressure sensors, this research proposes a fabrication and performance test of an SiC based high-temperature pressure sensor. By combining the technology of ICP etching, high temperature rapid annealing and femtosecond laser deep etching, an SiC high-temperature pressure sensor was developed and tested. Experimental conclusions are as follows:

(1) Using a femtosecond laser is a feasible method to overcome the problem of SiC deep etching as the developed SiC pressure sensor’s accuracy can reach 0.33%FS;

(2) The developed sensor can withstand high and low temperatures from −50 °C to 600 °C, which has exceeded the current operating temperature limit of silicon-based high-temperature pressure sensors.

Future research will focus on femtosecond laser processing parameter improvement, as well as the combination of femtosecond laser processing with MEMS (micro-electromechanical system) technology in mass manufacturing.

## Figures and Tables

**Table 1:** Process parameters of femtosecond laser etching.

Parameters	First Step Etching	Second Step Etching
Laser wavelength	1030 nm	
Repetition frequency	100 kHz	
Pulse width	240 fs	
Laser delay	150 μs	
Laser off delay	120 μs	
Poly delay	5 μs	
End delay	5 μs	
Laser power	5 W	4.2 W
Number of scans	3000	1200
Scanning speed	2500 mm/s	3000 mm/s
Scanline spacing	10 μm	5 μm
Etching depth	200 μm	50~70 μm

**Table 2:** Sensor’s output voltage in static calibration.

Pressure	1st Calibration	2nd Calibration	3rd Calibration	4th Calibration	5th Calibration
0 MPa	−0.01 mV	−0.01 mV	−0.02 mV	−0.02 mV	−0.03 mV
1 MPa	9.33 mV	9.32 mV	9.32 mV	9.31 mV	9.30 mV
2 MPa	18.92 mV	18.91 mV	18.90 mV	18.89 mV	18.89 mV
3 MPa	28.76 mV	28.76 mV	28.74 mV	28.73 mV	28.72 mV
4 MPa	38.87 mV	38.86 mV	38.85 mV	38.84 mV	38.83 mV
5 MPa	49.26 mV	49.24 mV	49.23 mV	49.22 mV	49.21 mV

**Table 3:** Static performance index comparison.

Reference	Repeatability Error	Hysteresis Error	Linearity Error
This paper	0.10%FS	0.11%FS	1.01%FS
[25]	0.23%FS	0.17%FS	0.03%FS
[30]	/	≤0.1%FS	0.49%FS
[31]	/	/	1.05%FS
[32]	/	0.17%FS	0.17%FS

**Table 4:** Thermal zero offset drift coefficient (TCO) of the sensor.

t (°C)	100	200	300	400	500	600
*TCO*	0.01%	0.03%	0.04%	0.06%	0.02%	0.04%

## Data Availability

The data presented in this study are available on request from the corresponding author. The data are not publicly available due to privacy.
